# Hereditary Leiomyomatosis and Renal Cell Cancer: Recent Insights Into Mechanisms and Systemic Treatment

**DOI:** 10.3389/fonc.2021.686556

**Published:** 2021-05-25

**Authors:** Congwang Zhang, Lijun Li, Yipeng Zhang, Changchun Zeng

**Affiliations:** ^1^ Department of Medical Laboratory, Shenzhen Longhua District Central Hospital, Shenzhen, China; ^2^ Department of Quality Control, Shenzhen Longhua District Central Hospital, Shenzhen, China; ^3^ Clinical Laboratory, Shenzhen Longhua District Central Hospital, Shenzhen, China

**Keywords:** hereditary leiomyomatosis and renal cell carcinoma, fumarate hydratase, pathogenesis, mechanism, treatment

## Abstract

Hereditary leiomyomatosis and renal cell carcinoma (HLRCC) is a rare autosomal dominant hereditary cancer syndrome characterized by a predisposition to cutaneous leiomyomas, uterine leiomyomas, and renal cell carcinoma (RCC). It is known to be caused by germline mutations of the fumarate hydratase (FH) gene, which encodes an enzyme component of the citric acid cycle and catalyzes the conversion of fumarate to L-malate. Currently, there is no standardized treatment for HLRCC, which may be due in part to a lack of understanding of the underlying mechanisms. Here, the underlying molecular mechanisms by which the inactivation of FH causes HLRCC are discussed. Additionally, potential therapeutic pharmacological strategies are also summarized to provide new perspectives for the prevention and treatment of HLRCC.

## Introduction

Hereditary leiomyomatosis and renal cell cancer (HLRCC), also known as leiomyomatosis and renal cell cancer (LRCC), is a rare autosomal dominant disorder in which the affected individuals have a higher risk of developing cutaneous leiomyomas, uterine leiomyomas, and renal cell carcinoma (RCC) (1). HLRCC, estimated to have an estimated incidence of approximately 1 in 200,000, is caused by inactivating mutations in the fumarate hydratase (FH) gene (2–6).

Renal cell carcinoma in HLRCC was previously classified as type 2 papillary renal cell carcinoma. HLRCC is currently recognized as epithelial renal tumors in the 2016 Worldwide Health Organization (WHO) classification of renal tumors (3, 7, 8). The penetrance of renal cancer in HLRCC is 10-20%. HLRCC tends to occur at an early age of onset and presents an aggressive clinical behavior with a poor prognosis (3, 9). There is a tendency to develop into uterine and cutaneous leiomyomas as well as papillary RCC in individuals with HLRCC, and approximately 15% to 30% of HLRCC patients develop kidney cancer (4–6, 10, 11). According to the latest analysis of 672 HLRCCs, 71.5% of patients had skin leiomyomas, 83% of women had uterine leiomyomas, and 34.9% of patients had RCCs (1). Furthermore, uterine leiomyomas appeared in 79% to 100% of women carrying the FH mutation (6, 11, 12).

A portion of patients with HLRCC are probably misdiagnosed as papillary RCC and treated following papillary RCC therapy (12). In addition, patients diagnosed with advanced HLRCC lack effective treatment strategies (13). There is a current shortage of FDA-approved medications for HLRCC. HLRCC is classified as non-clear cell carcinoma, of which the subtypes are diverse, difficult to diagnose, and relatively infrequent. In NCCN guidelines and clinical practice, patients with HLRCC will be treated with conventional clear cell carcinoma medications, such as vascular endothelial growth factor receptor (VEGFR) tyrosine kinase inhibitors (TKIs), mammalian target of rapamycin (mTOR) inhibitors, and immune checkpoint inhibitors (14, 15). However, clear cell renal carcinoma (ccRCC) and non-RCC have completely different genetic characteristics, and different pathological types of no-RCC have different clinical characteristics and drug responses (16). As a result, conventional medications for the treatment of ccRCC are not particularly effective for HLRCC, and alternative treatment options for HLRCC are urgently needed. Elucidation of the underlying mechanisms involved in the progression of HLRCC may reveal underlying therapeutic targets that may provide new information on the management of HLRCC (5, 17).

The FH gene located at 1q42.3-q43 encodes an enzyme component involved in the tricarboxylic acid (TCA) cycle, which facilitates the formation of L-malate from fumarate (18, 19). FH inactivation results in decreased oxidative phosphorylation and metabolic reprogramming to aerobic glycolysis, also known as the “Warburg effect” (20, 21). The mutated FH eliminates the hypoxia-inducible factor (HIF) prolyl-hydroxylase and enhances the hypoxia-inducible factor 1 alpha (HIF1alpha), which further affects downstream effectors, such as glucose transporter 1, erythropoietin, and vascular endothelial growth factor, and induces epigenetic changes, causing cell proliferation, metastasis, and tumor. In addition, the inactivation of FH contributes to the accumulation of fumarate, which increases oxidative stress and stimulates the signaling pathway of NRF2-antioxidant response elements (14, 17, 22).

The purpose of this study is to summarize recent advances in the molecular mechanism of HLRCC and to describe the potential treatment strategies for HLRCC to assist clinicians in managing HLRCC.

## Pathogenesis

### FH Inactivation and the Activation of HIF

**Figure 1:**
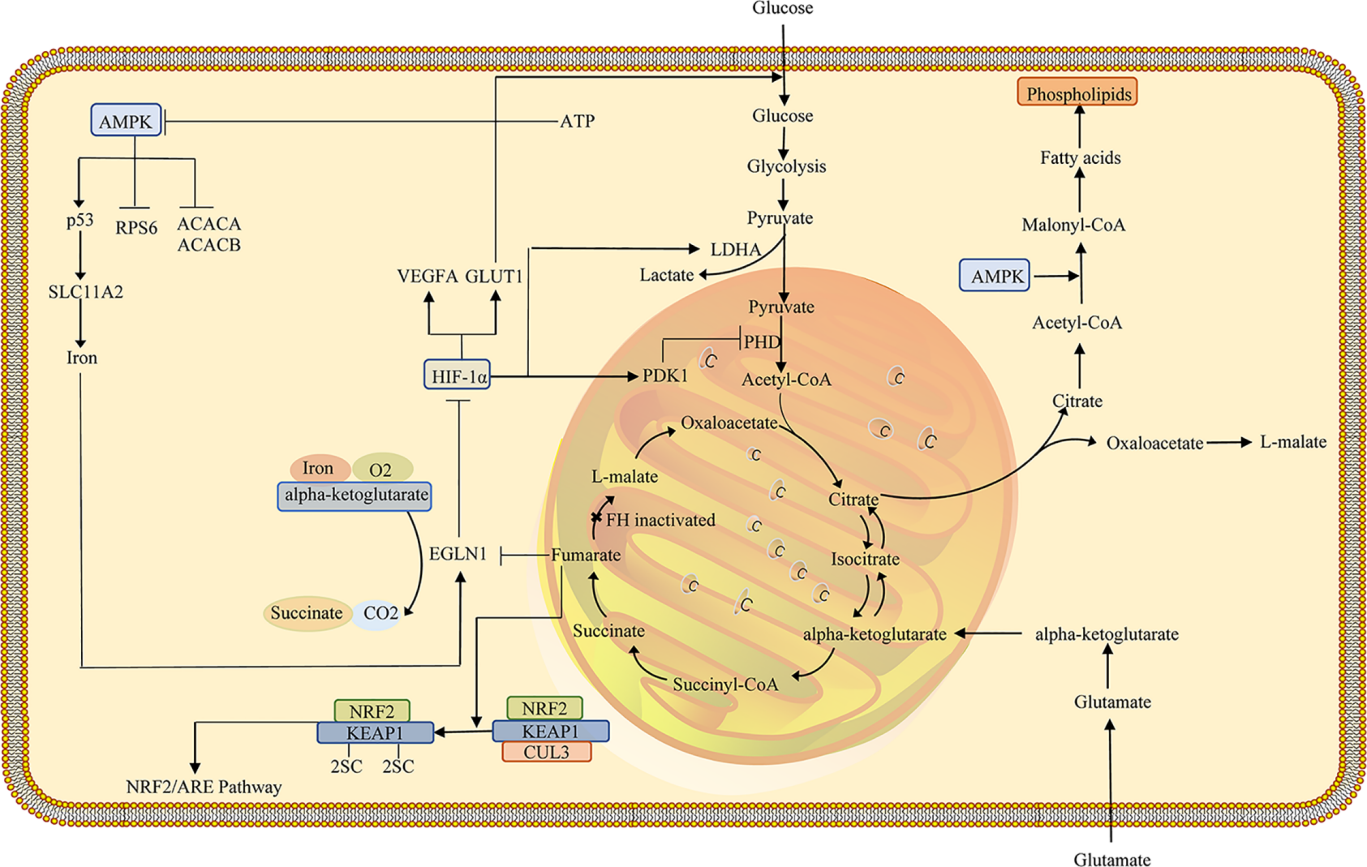
Schematic representation of HLRCC pathway. HLRCC, marked by defective oxidative phosphorylation, undergoes a metabolic transition to aerobic glycolysis to produce ATP required for the energetic requirements of proliferating cells. Enhanced glycolysis represses AMPK expression and activation, resulting in increased S6 and ACC activity, fostering anabolic growth and proliferation. Decreased AMPK yields decreased p53 and iron transporter DMT1. Cytosolic iron concentrations will suppress prolyl hydroxylase, which is susceptible to iron levels, stabilizing HIF1α. Fumarate, which rises in FH-deficient cells, has been shown to suppress prolyl hydroxylase, which will contribute to more stabilization of HIF1α, enhancing the transcription of factors such as VEGF and GLUT1. Enhanced fumarate was shown to succinate KEAP1, thereby altering its conformation and affecting its capacity to cause Nrf2 degradation. Nrf2 transcription activates anti-oxidant reactions and protects against oxidative stress. Increased HIF1α will stimulate LDHA, increase lactate development, and stimulate PDK1, which inhibits PHD and decreases pyruvate entry into the TCA cycle. HLRCC utilizes glutamine-dependent reductive carboxylation to form citrate. HLRCC, hereditary leiomyomatosis and renal cell carcinoma; FH, fumarate hydratase; AMPK, AMP-activated protein kinase; PHD, prolyl hydroxylase; PDK1, pyruvate dehydrogenase kinase 1.

FH inactivation was confirmed to be associated with the initial tumor-promoting event in HLRCC, leading to decreased FH enzyme function. As shown in **Figure 1**, FH inactivation contributes to the activation of the hypoxia-inducible factor (HIF), which is generally comprised of α and β subunits and functions as an oxygen sensor (23). Under physiological oxygen levels, HIF prolyl hydroxylases (PHDs) work with oxygen (O2), 2-oxoglutarate (2-OG), ascorbic acid, and iron (Fe2+) to hydroxylate specific proline residues within the oxygen-dependent degradation domain (ODD) of HIF-α subunits, causing HIF ubiquitination. Subsequently, HIF is recognized by von Hippel-Lindau tumor suppressor protein (pVHL) and degraded by the proteasome (24, 25).

In tumor cells, HIF activation is incredibly essential to coordinate its adaptation to hypoxic conditions (26, 27). HIF-1 regulates metabolic transformation by modulation of the activity of GLUTs participants in glycolysis. In glycolysis, glucose is ultimately converted to pyruvates, ATP, and NADH, and the pyruvate is subsequently applied in the tricarboxylic acid cycle or works as a precursor implicated in other reactions (28). GLUTs contribute to glucose absorption and accelerate the metabolic shift towards glycolysis in glucose metabolism, causing increased glucose absorption in hypoxic cancer cells (29–31). In hypoxia, HIFα is incapable of being hydroxylated and evades pVHL recognition, which causes HIFα to accumulate. HIFα migrates to the cellular nucleus and subsequently interacts with HIF-1β to form a HIF-complex, which binds to the DNA and initiates a transcriptional program to counter hypoxia (14, 32, 33). HIF-1α can bind with enhancers or promoters of the target genes and activate and modulate the transcription of target genes. Various target genes, such as vascular endothelial growth factor (VEGF), erythropoietin (EPO), epidermal growth factor (EGF), transforming growth factor beta3 (TGF-β3), and glucose transporters (GLUTs) are critical to cancer development (29, 33). HIF-1 acts as a transcription factor of VEGF and supports vessel formation, which is essential for tumor growth, and angiogenesis (34–36). Additionally, inhibiting HIF-1 is responsible for the decreased secretion of VEGF and suppresses tumor growth.

The decreased activity of prolyl hydroxylases induces the accumulation of HIF-1α. Loss of FH can induce reactive oxygen species (ROS) by inhibiting the activity of prolyl hydroxylases, which may contribute to the stabilization of HIF-1α (10). Antioxidants can inhibit increases in ROS, and the high levels of ROS may lead to constitutive activation of HIF. Moreover, HIF activation can not only eliminate the formation of ROS by blocking the TCA cycle but also enhance ROS formation *via* NADPH oxidase (37–39). ROS involved in tumor formation accumulates in tumors with FH-deficient, and high levels of ROS cause the accumulation of HIFα (23, 40, 41). Iron oxidation is the mechanism by which ROS stabilizes HIF. Ferrous iron (Fe2+) was oxidized in ferric form (Fe3+) with hydrogen peroxide, and Fe2+ loses the ability to bond with PHDs, which is crucial for HIF labeling for VHL-mediated HIF ubiquitination and proteasomal degradation (42, 43). Additionally, HIF PHD inhibitors enhance HIF concentrations and reduce ROS formation, indicating the potential therapeutic value of HIF PHD inhibitors (38).

### Epithelial to Mesenchymal Transition

**Figure 2:**
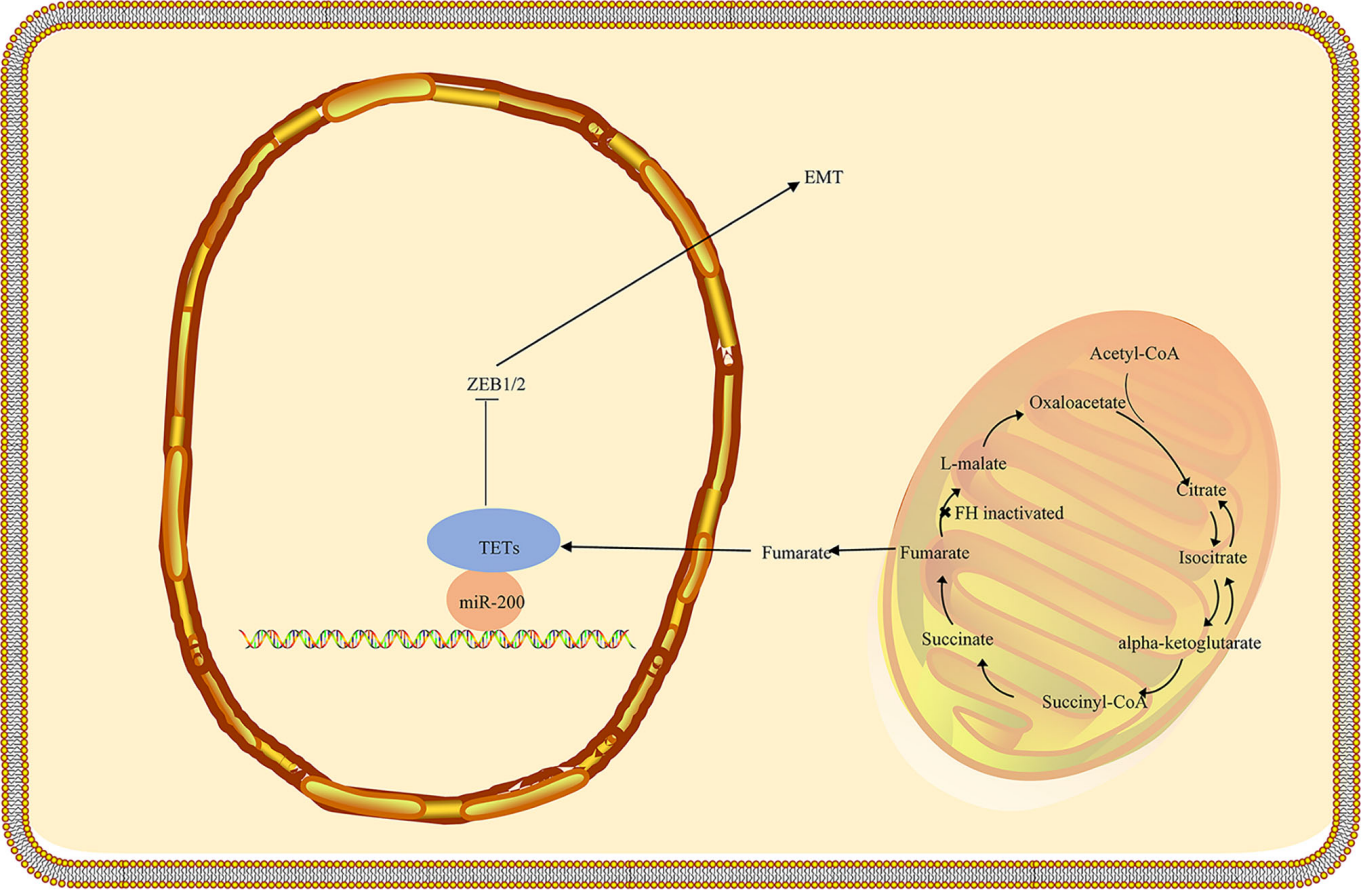
Schematic representation of the potential association between FH loss and EMT activation in HLRCC. The TET3-mediated demethylation of the miR-200 cluster is blocked as fumarate accumulates as a consequence of FH inactivation, resulting in epigenetic repression of the miR-200ba429 cluster. As a result, Zeb1/2 is triggered, initiating a signaling cascade that contributes to EMT. FH, fumarate hydratase; EMT, Epithelial-mesenchymal transition; HLRCC, hereditary leiomyomatosis and renal cell carcinoma.

Epithelial to mesenchymal transfer (EMT) refers to the transformation of epithelial cells into mesenchymal cells. EMT results in epithelial cells losing their adhesion after polarization and acquiring the mesenchymal cell phenotype. The EMT process is involved in a variety of metabolic pathways, such as the TCA cycle, glycolysis, and lipid metabolism, associated with the development and progression of cancer (44–46). In addition to metabolic disruption, fumarate accumulation appears to reinforce EMT, a pro-oncogene and pro-metastatic process by which epithelial cells are converted into mesenchymal cells (**Figure 2**) (47). As previously mentioned, fumarate can suppress HIF prolyl hydroxylases, members of the dioxygenase family, utilizing 2-OG and oxygen as co-substrates, as well as ascorbate and iron as cofactors (48). In addition, Ten-Eleven Translocation (TET) enzymes that are involved in epigenetic regulation can catalyze the oxidation of 5-methylcytosine to 5-hydroxymethylcytosine oxidized (5-hmC), leading to the demethylation of DNA (49, 50). Fumarate acts as a TETs inhibitor that can reduce the levels of 5-hmC, indicating the underlying epigenetic effect of fumarate (51). FH-deficient cells exhibit mesenchymal characteristics. Fumarate induces EMT in FH-proficient cells. FH deficiency is associated with an EMT signature in HLRCC. These findings indicate that dysregulation of FH activity and fumarate accumulation contribute to the induction of EMT and the aggressive characteristics of FH-deficient tumors (47, 52). Vimentin mediates the expression of various transcription factors related to EMT in FH-deficient cells, which may be inhibited by miRNAs, such as the miR-200 family (53). Furthermore, miR-200 was found to be extensively regulated downwards in FH-deficient cells, consistent with the consensus of previous studies that methylation of miRNA-200 in the promoter region is modulated by TET demethylation (44, 47, 54). The results imply that FH loss and fumarate accumulation exert a vital part in EMT induction, leading to hypermethylation and miR-200 inhibition (47).

### DNA Damage Repair

**Figure 3:**
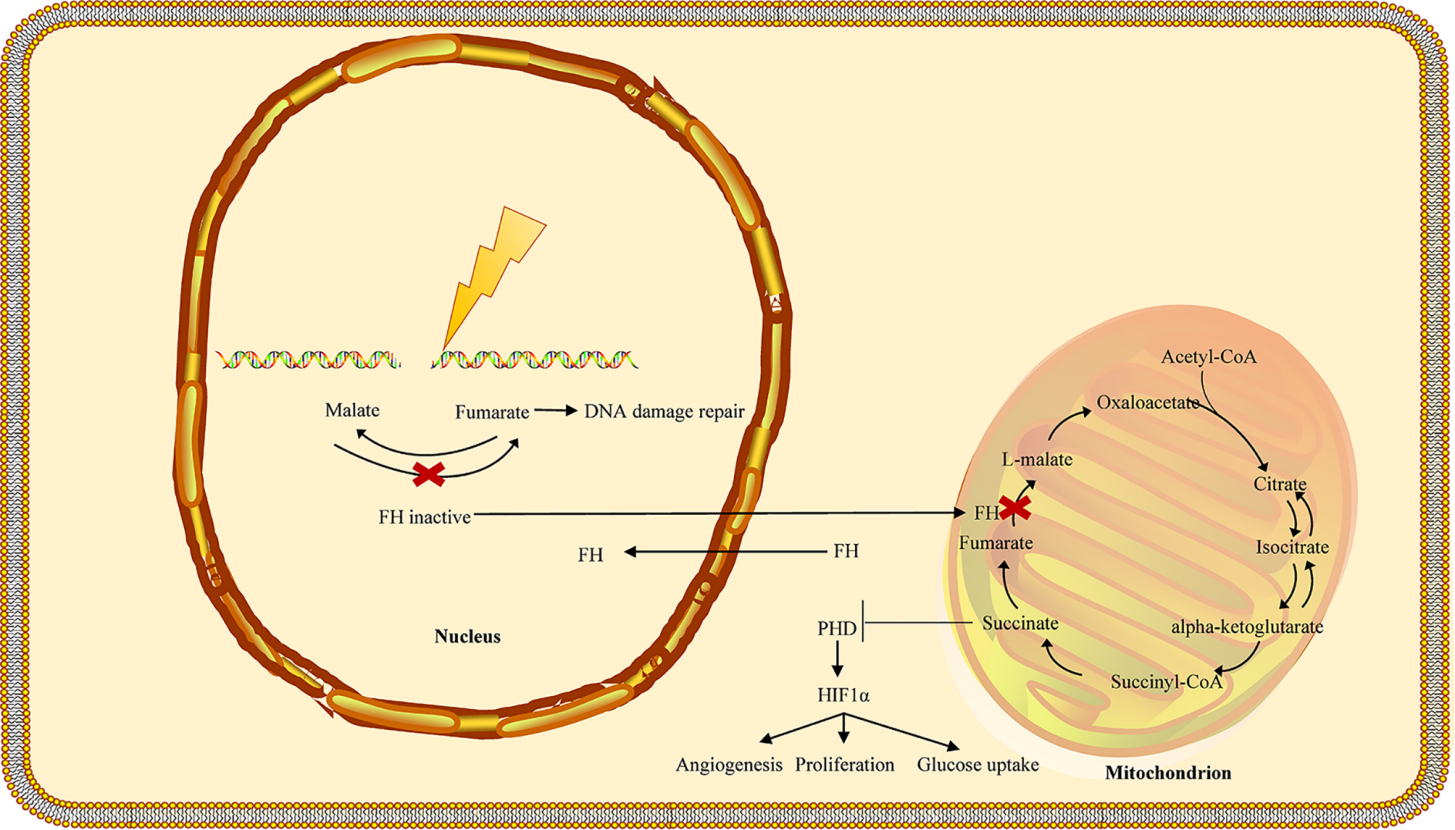
FH loss is involved in the DNA damage response in HLRCC. In the nucleus, fumarate accumulation increases the resistance to DNA damage by ionizing radiation (IR) and promotes the non-homologous end-joining upon DNA damage by inhibiting KDM6, a key histone of demethylase involved in the deployment of chromatin for DNA repair. Biallelic inactivation of fumarase in a single tumor cell may prevent the cell from producing fumarate in close proximity to the cellular DNA, lowering genomic stability and leading to the formation of new mutations. The fumarate concentration in a single tumor cell may not be sufficient to stabilize HIF. Furthermore, the proliferation of fumarase deficient cells may shape a tightly packed cell population, allowing fumarate levels to rise to the concentration needed for HIF stabilization. FH, fumarate hydratase; HLRCC, hereditary leiomyomatosis and renal cell carcinoma.

Recent research suggests that fumarase and its associated metabolite fumarate are involved in the cellular DNA damage response. The biallelic inactivation of fumarase can suppress fumarate generation and decrease genomic stability (55, 56). Ionizing radiation induces phosphorylation of FH and stimulates its binding to the DNA double-strand break (DSB) region, which can bind to the DNA-dependent protein kinase (DNA-PK) holoenzyme and cause DNA-PK catalytic subunit (DNA-PKcs) activation in non-homologous end joining (NHEJ) (57, 58). Accumulated fumarate generation suppresses the activity of KDM2B in DSB regions, elevates H3K36me2 levels, and accumulates DNA-PK complex in DSBs for NHEJ (59, 60). DNA-PK–mediated fumarase phosphorylation facilitates fumarate-induced DNA repair by demethylation of histone H3, suggesting the significant role of FH in repairing DNA damage (**Figure 3**) (58, 61–63). The fumarate accumulated in HLRCC inhibits homologous recombination (HR) and accelerates double-stranded DNA breaks by suppressing lysine demethylase, KDM4B, a member of the KMD4 family of histone demethylase, closely associated with DNA damage (61, 64). Additionally, FH loss is responsible for the resistance to ionizing radiation and motivates the early mitotic entry after ionizing radiation. Moreover, fumarate accumulation facilitates G2-M transition and causes a decrease in the G2 checkpoint, which tends to damage the DNA and confers resistance to ionizing radiation in FH-deficient cells, indicating that FH loss and fumarate accumulation can give rise to genomic instability that can trigger HLRCC formation (58).

### The KEAP1-NRF2 Pathway in FH-Deficient Kidney Cancer

**Figure 4:**
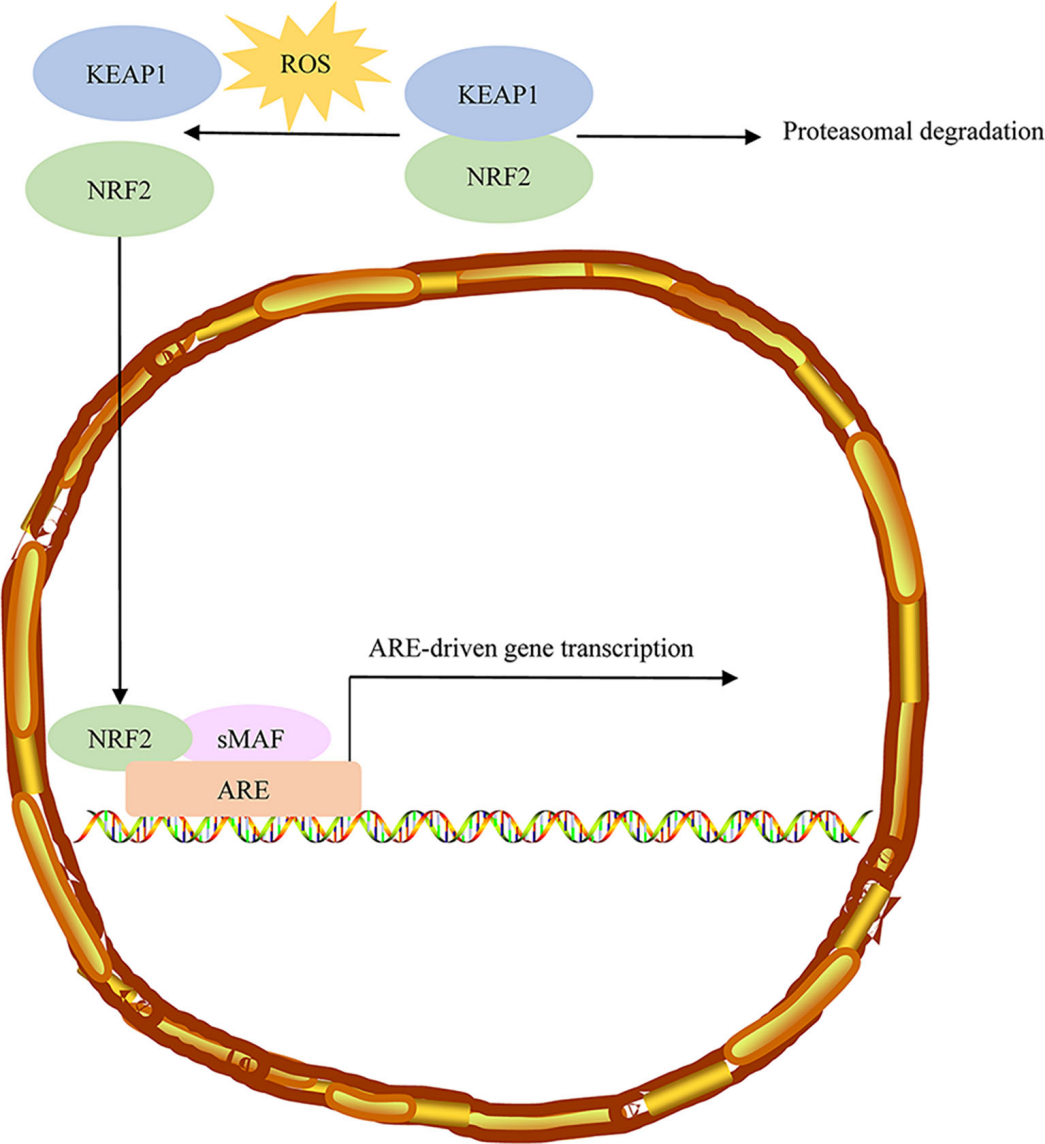
Sustained NRF2 activation in HLRCC. Under normal conditions, NRF2 associates with the Cul3-Rbx1 E3 ubiquitin-ligase substrate KEAP1, which keeps NRF2 primed for ubiquitination and proteasomal degradation. ROS stress induces conformational changes in KEAP1, resulting in NRF2 dissociation. Free NRF2 reaches the nucleus, where it forms dimers with small MAF proteins and binds to AREs regulatory sequences of target genes. NRF2, Nuclear factor erythroid 2-related factor 2; HLRCC, hereditary leiomyomatosis and renal cell carcinoma; AREs, antioxidant responsive elements.

Some studies have suggested that the irreplaceable role of the Kelch-like ECH-associated protein 1 (KEAP1)/nuclear factor E2-related factor 2 (Nrf2) pathway in tumor metabolism (65). Loss of enzymatic activity in fumarate hydration results in fumarate accumulation and further to the succination of Keap1 cysteine residues (2SC) (66). Previous studies have shown that the high level of fumarate accumulated in HLRCC tumor cells causes aberrant succination of cellular proteins by forming a stable chemical modification, S-(2-succino)-cysteine (2SC), detectable by immunohistochemistry. Most confirmed HLRCC tumors showed diffuse and strong staining of nuclear and cytoplasmic 2SC, while all clear cells, most high-grade unclassified RCC, and the large majority of type 2 papillary cases showed no 2SC immunoreactivity (67). FH and 2SC are two highly correlated immunohistochemical biomarkers in the HLRCC diagnosis (68). NRF2, a member of a small family of basic leucine zipper (bZIP) proteins, encoded by the nuclear factor, erythroid derivative two like 2 (NFE2L2) gene, is a transcription factor that affects oxidative and electrophilic stress. NRF2 can bind to antioxidant response (ARE) elements in the promoter region of various genes (69). Accumulating evidence suggests that the controlled activation of NRF2 can eliminate reactive-oxygen species (ROS) and weaken DNA damage, thereby reducing the probability of cancer onset and progression in normal cells. However, in transformed cells, activation of NRF2 signaling is associated with an unfavorable prognosis and leads to malignant progression (**Figure 4**) (70). Under normal conditions, KEAP1 regulates the stability of the NRF2 protein through proteasomal degradation. NRF2 interacts with the KEAP1 complex in the cytoplasm, and NRF2 is continuously ubiquitinated and degraded to ensure low levels of NRF2 and avoid the activation of its target genes. Under stressed conditions, oxidative/electrophilic stress induces a conformational change in the KEAP1 complex and contributes to the dissociation of the NRF2 from KEAP1 and the translocation of the NRF2 into the nucleus. The NRF2 accumulated in the nucleus forms heterodimerization with small MAF proteins and further binds to the ARE components of the target genes and promotes the expression of the target genes (71).

The target genes of Nrf2 possess antioxidant properties and motivate the cellular reaction to eliminate xenobiotics. Multiple antioxidant enzymes such as peroxiredoxin 1 (Prdx1), heme oxygenase-1 (HO-1), superoxide dismutase-1 (SOD-1), and NAD(P)H dehydrogenase quinone 1 (NQO1) and various enzymes such as glutamate-cysteine ligase modifier (GCLM) and glutathione S-transferases (GSTs) participate in the synthesis of glutathione. Additionally, the target genes of Nrf2 have cytoprotective properties, exhibiting anti-inflammatory activity, and accelerating the degradation of proteins oxidatively damaged by the proteasome. Activating Nrf2 facilitates Nrf2 expression and accelerates cellular stress reactions (72–74). Furthermore, fumarate accumulation may contribute to regulating the stability of NRF2 by glutathione succination. Moreover, NRF2 activation activated by FH loss is a result of oxidation-reduction imbalance attributed to glutathione succination in FH-deficient cells (75). Sustained activation of NRF2 has been observed in HLRCC, caused by the accumulation of fumarate, which may be attributable to FH inactivation (76). Moreover, somatic mutations in NRF2, SIRT1, and CUL3 may activate NRF2 and affect NRF2 transcription activity (77).

## Systemic Treatment of HLRCC

### Bevacizumab and Erlotinib

In HLRCC, HIF accumulation leads to the disruption of multiple signaling pathways associated with malignant progression. FH inactivation causes VHL-independent HIF upregulation and NRF2 activation. Therefore, targeting these critical downstream pathways or the pivotal pathway components, such as vascular endothelial growth factor (VEGF), transforming growth factor-alpha (TGF-α), and epidermal growth factor receptor (EGFR) may inhibit tumorigenesis.

Erlotinib, an inhibitor of EGFR tyrosine kinase, has been approved by FDA for the treatment of advanced or metastatic non-small cell lung cancer patients with EGFR exon 19 deletions or exon 21 (L858R) substitution mutations. Bevacizumab, a VEGF inhibitor, is widely used in a variety of cancers, such as non-small cell lung cancer, renal cell carcinoma, cervical cancer, peritoneal cancer, epithelial ovarian cancer, fallopian tube cancer, and glioblastoma.

A phase II clinical trial of bevacizumab (a humanized anti-VEGF monoclonal IgG1 antibody) plus erlotinib (an inhibitor of EGFR tyrosine kinase) in patients with HLRCC (AVATAR trial, https://clinicaltrials.gov/ct2/show/NCT01130519) exhibited that the objective response rate (ORR) was 65% and the median progression-free survival (PFS) was 24.2 months in patients with HLRCC. According to this impressive study, the National Comprehensive Cancer Network (NCCN) guidelines recommended this treatment regimen to treat HLRCC (78). Additionally, a recently updated AVATAR trial result indicated that the ORR was 64% (27/42; 95% confidence interval [CI], 49 to 77), and the median PFS was 21.1 months in type 2 papillary RCC subgroup associated with HLRCC (79).

A real-world outcome of the combination of bevacizumab plus erlotinib subjects in South Korea with HLRCC exhibited that the ORR was 50% (5/10; 95% CI, 24 to 76) and the disease control rate was 90% (9/10; 95% CI, 60 to 98) (78). Besides, after treatment failure with axitinib, bevacizumab plus erlotinib received a sustained response of over18 months in a patient with locally advanced HLRCC (80). In a nutshell, the combination of bevacizumab and erlotinib may be an effective treatment for advanced HLRCC (**Table 1**).

**Table 1:** Summary of the underlying therapeutic strategies.

Underlying therapeutic strategies	Targets	References
Bevacizumab and Erlotinib	VEGF; EGFR	(78–80)
PARP Inhibition	PARP	(61)
Vandetanib and Metformin	EGFR, VEGFR, RET, BRK, TIES2, EPH, SRC; AMPK	(98)
Sintilimab and Axitinib	PD-1; VEGFR-1,-2,-3	(107)
Nivolumab and Axitinib	PD-1; VEGFR-1,-2,-3	(106)
Nivolumab and Ipilimumab	PD-1; CTLA-4	(108)
Cabozantinib	MET, VEGFR-1, -2, -3, AXL, RET, ROS1, TYRO3, MER, KIT, TRKB, FLT-3, and TIE-2	(110)

### PARP Inhibition

Synthetic lethality is a condition where simultaneous deficiencies in the two or more genes from complementary pathways lead to the death of cancer cells. The genetic interaction between members of the BRCA and PARP pathway can be depicted as synthetic lethality. Mutations in the members of the BRCA pathway lead to a defective HR pathway, causing tumor cell injury in the presence of PAPR inhibitors. If mutations in the BRCA genes cause loss of function of the BRCA1 and BRCA2 proteins, they cause HRD (homologous recombinant deficit). Additionally, other HRR-related genes, such as PALB2, CDK12, RAD51, CHEK2, ATM mutations, or BRCA1 gene promoter methylation, and other obscure reasons will cause HRD and cause genome instability. PARP is a crucial protein in the base excision repair (BER) pathway, participated in the repair of DNA single-strand breaks (SSBs). When the BER pathway has undergone alterations, improperly repaired SSBs accumulate and degenerate to double-strand breaks (DSBs), resulting in increased dependence on other repair pathways, such as homologous recombination (HR) and the non-homologous end joining (NHEJ) (81, 82). Recombinant homologous repair (HRR) is an important way to repair double-strand DNA damage. HRR is a complex multi-step signal pathway, including key proteins BRCA1 and BRCA2 (83–85).

The FDA has approved several PARP inhibitors, such as rucaparib, olaparib, niraparib, and talazoparib for the treatment of advanced cancers carrying BRCA1/2 mutations, mainly including breast, ovarian, fallopian tube, or primary peritoneal, prostate, and pancreatic cancer. In terms of mechanism of action, tumor cells with homologous recombinant defect (HRD) are more sensitive to platinum medicines or PARP inhibitors. However, it is not sufficient to use BRCA1/2 as the HRD classification standard, as there are already dozens of genes involved in HRR, and abnormalities of these genes can also lead to the HRD phenotype (86, 87). In October 2019, the FDA approved niraparib for patients with advanced ovarian, fallopian tube, or primary peritoneal cancer treated with three or more prior chemotherapy regimens and whose cancer is associated with positive HRD status. HRD is defined as either a deleterious or suspected BRCA mutation or genomic instability in patients whose disease progression is greater than six months after the response to the last platinum chemotherapy.

As previous research has shown, FH loss is closely associated with DNA damage repair, indicating a potential clinical benefit of poly (ADP-ribose) polymerase (PARP) inhibitors in HLRCC treatment (58). Ionizing radiation, etoposide, mitomycin C, and cisplatin as DNA damaging agents can cause DNA damage and inhibit the survival of the FH-deficient cells. Moreover, FH loss, as well as fumarate intake, enhanced sensitivity to the PARP inhibitors, such as BMN-673, and olaparib, which are known to affect homologous recombination deficiency (HRD) tumors. Furthermore, elevated sensitivity to PARP inhibitors has been discovered in both HLRCC cell models and tumor xenograft models. Excessive production of fumarate in HLRCC undermines homologous recombination (HR) and makes FH-deficient cells highly susceptible to PARP inhibitor (61).

Recent work has highlighted that elevated fumarate leads to G2 checkpoint abrogation and further increases endogenous damage. The error-prone non-homologous end-joining (NHEJ) and continuous cell divisions further cause genomic instability and increase susceptibility to the combination of kinase inhibitors PARP and WEE1 which may suppress the G2-M checkpoint arrest and improve the therapeutic efficacy of olaparib (88). Briefly, preclinical studies have shown that PARP inhibitors are promising therapeutic agents in HLRCC treatment, but more clinical evidence is required to support this conclusion.

### DNA Hypermethylation

In FH-deficient cells, the accumulated fumarate leads to the suppression of the Ten-eleven translocation (TET) enzymes which are 2-oxoglutarate (2-OG)-dependent dioxygenases. TET enzymes, catalyzing the oxidation of 5-methylcytosine DNA (5 mC) to 5-hydroxymethylcytosine (5hmC), accelerate the demethylation of DNA that may affect transcriptional control. DNA methylation, a common epigenetic modification, is widely known to facilitate tumorigenesis (89, 90). DNA methyltransferases (DNMT) are responsible for the methyl-transfer reaction, which catalyzes the transfer of S-adenosyl methionine (SAM) methyl groups to DNA and modifies gene transcription. DNMT inhibitors suppress DNA methylation and mitigate the impact of abnormal epigenetic changes in cancer (91–94). SGI-110 (guadecitabine), a second-generation DNA hypomethylating agent composed of endogenous nucleoside deoxyguanosine and decitabine, is specially designed to alleviate degradation by protein aminases and increase the effect of the active decitabine metabolite on tumor growth (95, 96). A phase II study (https://clinicaltrials.gov/ct2/show/NCT03165721) designed to assess the clinical value of guadecitabine in patients with HLRCC showed that the enrolled HLRCC patient did not receive a complete or partial response. Another patient with HLRCC died as a result of disease progression (97). In general, the clinical activity and toxicity of guadecitabine in HLRCC seem unsatisfactory.

### Vandetanib and Metformin

Vandetanib, a selective inhibitor of the vascular endothelial growth factor receptor (VEGFR), epidermal growth factor receptor (EGFR), and Ret Proto-Oncogene (RET) tyrosine kinases, is widely used in treating patients with advanced or metastatic medullary thyroid cancer. Both the EGFR- and VEGFR-dependent pathways are identified as one of the critical processes in tumor growth and metastasis. Activation of RET receptor tyrosine kinase (RTK) triggers multiple signaling pathways, suggesting that inhibition of RET activity may delay tumor progression. As described in previous studies, vandetanib has been shown to inhibit ABL1 activity that demonstrated synthetic lethality with FH (98, 99). ABL proto-oncogene 1, Non-Receptor Tyrosine Kinase (ABL1) is a member of the ABL family of non-receptor tyrosine kinases implicated in multiple cellular processes, such as cell motility and adhesion, DNA damage response, autophagy, and differentiation (100, 101). In UOK262 cells, vandetanib, as inhibition of ABL1, is responsible for a gradual decrease in HIF1α expression and mammalian target of rapamycin (mTOR) activity, and the cells sensitive to vandetanib are dependent on ABL1 activity. Moreover, elevated expression of ABL1 was recognized in human tumor samples compared to normal renal tissue. Also, ABL1 contributes to aerobic glycolysis, and vandetanib can inhibit glycolysis and delay tumor progression. Besides, the inhibition of ABL1 appears to facilitate the transfer of NRF2 from the nucleus to the cytoplasm and lead to diminished transcription of its target genes, indicating a vital role of ABL1 in the regulation of the KEAP1/NRF2 pathway.

AMP-activated protein kinase (AMPK) activator metformin reduced NRF2 acetylation, and the combination of metformin and vandetanib resulted in an additive inhibition of NRF2 in UOK262 cells. Compared with the administration of a single vandetanib agent, the addition of a clinically achievable dose of metformin decreased the effective concentration of vandetanib by 90% in an HLRCC xenograft model. Metformin regulates AMPK, vandetanib inhibits ABL1, and the combination of metformin and vandetanib may inhibit NRF2 activity to the maximum extent *in vitro* (98). In UOK262 cells, the attenuation of AMPK activity causes metabolic alternations. Activating AMPK facilitates the expression of the silent information regulator T1 (SIRT1) in a vandetanib independent manner, leading to the deacetylation and suppression of NRF2. A combination of vandetanib and metformin contributes to a decrease in the KEAP1/NRF2 pathway activity in HLRCC (98). Besides, a phase I/II trial (https://clinicaltrials.gov/ct2/show/NCT02495103) of vandetanib in combination with metformin in subjects with HLRCC is underway, and the effectiveness of this therapeutic strategy deserves to be evaluated.

### Checkpoint Inhibitor Immunotherapy

The United States Food and Drug Administration (FDA) has approved PD-1 inhibitors (nivolumab and pembrolizumab) and PD-L1 inhibitors (atezolizumab, avelumab, and durvalumab) in combination with other regimens for metastatic RCC. However, there is a current shortage of FDA-approved medications for HLRCC. HLRCC is classified as non-clear cell carcinoma, which has a variety of subtypes that are difficult to diagnose and relatively low incidence. In the NCCN guidelines and actual clinical practice, patients with HLRCC will be treated with drugs against clear cell carcinoma. Importantly, there are some case reports of immune checkpoint inhibitors showing promising results, and clinical trials involving immune checkpoint inhibitors are underway, suggesting that immune checkpoint inhibitors may be a promising therapeutic target in HLRCC. Therefore, for advanced or metastatic HLRCC patients, participating in relevant clinical trials is a treatment strategy that may be considered. However, there is minimal information on the potential therapeutic implications of immune checkpoint inhibitors in the treatment of patients with HLRCC (102–104).

Recently, the expression of PD-1/PD-L1 in 13 HLRCC is assessed for the first time using immunohistochemistry (IHC) and quantitative polymerase chain reaction (qPCR), and the results have shown that PD-1/PD-L1 expression is limited to a small proportion of HLRCC patients, who are more likely to benefit from immunotherapy (105). Moreover, a single-arm phase II clinical trial (https://clinicaltrials.gov/ct2/show/NCT04146831) to assess the safety and efficacy of sintilimab (a PD-1 inhibitor) as second-line treatment in HLRCC is going. Besides, a single-arm phase II clinical trial (https://clinicaltrials.gov/ct2/show/NCT04387500) to assess the safety and efficacy of sintilimab (a PD-1 inhibitor) in combination with axitinib (a VEGFR1/2/3 inhibitor) in HLRCC is also currently underway. Furthermore, one case of metastatic HLRCC achieved long-term survival with cytoreductive nephrectomy, followed by a sequence of axitinib and nivolumab (106). Another case of recurrent and metastatic HLRCC achieved acceptable efficacy from the association of sintilimab and axitinib (107). The combination of nivolumab (a PD-1 inhibitor) and ipilimumab (an inhibitor of CTLA-4) demonstrates promising therapeutic perspectives in a variety of tumor types. The FDA has approved the combination of nivolumab and ipilimumab for the treatment of metastatic ccRCC. Additionally, after 31 weeks of combined nivolumab and ipilimumab, a patient with HLRCC achieved a complete response (108). The PD-1/PD-L1 interaction occurs primarily in surrounding tissues and organs and primarily plays a role in the effective stage of T cell activation. CTLA-4 is expressed by Treg cells and memory CD4+ cells and plays a role in the early activation of T cells in lymphoid tissues (109). Taken together, it seems possible to consider combining immunotherapy with a targeted treatment for HLRCC.

### Others

Cabozantinib has been approved for patients with metastatic renal clear cell carcinoma. However, studies on the safety and efficacy of cabozantinib in non-clear-cell RCC treatment are relatively limited, especially in HLRCC. A multicenter retrospective cohort study of 112 patients with advanced non-clear-cell renal cell carcinoma treated with cabozantinib was conducted to investigate the activity and toxicity of cabozantinib in the advanced non-clear-cell RCC. FH mutations were present in five patients with non-clear-cell RCC. Among them, one patient with collecting duct carcinoma and two patients with papillary cell carcinoma achieved a partial response, while the remaining two patients achieved stable disease, suggesting that cabozantinib seems to be effective for HLRCC (110).

2-deoxy-d-glucose (2DG; a glycolytic inhibitor) may stimulate AMPK and lead to AMPK-dependent mTORC1 suppression so that it was used to explore the treatment in a patient with HLRCC. Unfortunately, the patient died shortly after the 2DG treatment. Interestingly, sustained consumption of 2DG in the patient causes symptoms of hypoglycemia, indicating a significant inhibition of glycolysis (111). Further research is required to verify the feasibility of therapeutic strategies that disturb tumor glycolysis.

## Conclusions and Perspectives

HLRCC is a pathological type of biologically aggressive RCC. HLRCC is triggered by germline mutations in the FH gene that encodes the Krebs cycle enzyme. However, the molecular mechanisms by which FH mutations cause HLRCC are poorly understood. The accumulation of evidence on the molecular mechanisms will make it possible to better understand the pathogenesis of HLRCC. In HLRCC cells, FH loss leads directly to the accumulation of intracellular fumarate, resulting in the activation of multiple signaling pathway components, such as HIF1A, NRF2. Moreover, the accumulation of fumarate weakened the cellular homeostasis of iron. Besides, the accumulation of fumarate attenuated AMPK activity and facilitated a metabolic shift towards aerobic glycolysis.

Although there is still no standard treatment associated with HLRCC, multiple potential therapeutic approaches to address this early-onset disease are under consideration. Advances in pre-clinical and clinical research at HLRCC are summarized for the first time in this article. The combination of bevacizumab and erlotinib was recommended by NCCN for HLRCC treatment. Also, cabozantinib, a multikinase inhibitor that inhibits specific receptor tyrosine kinases such as VEGFR1, VEGFR2, VEGFR3, KIT, TRKB, FLT-3, AXL, RET, MET, and TIE-2, is an additional underlying option for HLRCC treatment. FDA-approved medications for renal cell carcinoma, such as sunitinib, everolimus, and pazopanib, are not satisfactory for HLRCC patients. Other frontline therapies for renal carcinoma, including ipilimumab combined with nivolumab, axitinib combined with pembrolizumab, and cabozantinib, seem to hold greater promise in HLRCC. Taken together, there will be more alternative treatment options moving forward with a deeper understanding of HLRCC.

## Author Contributions

All authors listed have made a substantial, direct and intellectual contribution to the work, and approved it for publication. All authors contributed to the article and approved the submitted version.

## Funding

This work was supported by the National Natural Science Foundation of China (81660755) and the Science and Technology Project of Shenzhen of China (JCYJ20170307160524377).

## Conflict of Interest

The authors declare that the research was conducted in the absence of any commercial or financial relationships that could be construed as a potential conflict of interest.

## Abbreviations

HLRCC, hereditary leiomyomatosis and renal cell carcinoma; RCC, renal cell carcinoma; FH, fumarate hydratase; LRCC, leiomyomatosis and renal cell cancer; WHO, Worldwide health organization; VEGFR, vascular endothelial growth factor receptor; TKIs, tyrosine kinase inhibitors; mTOR, mammalian target of rapamycin; TCA, tricarboxylic acid; HIF, hypoxia-inducible factor; PHD, prolyl hydroxylase; EPO, erythropoietin; EGF, epidermal growth factor; TGF-β3, transforming growth factor beta3; GLUTs, glucose transporters; O2, oxygen; 2-OG, 2-oxoglutarate; ODD, oxygen-dependent degradation domain; pVHL, Von Hippel-Lindau tumor suppressor protein; Fe2+, Ferrous iron; Fe3+: Ferric form; EMT: Epithelial-mesenchymal transition; TET: Ten-Eleven Translocation; 5-hmC: 5-hydroxymethylcytosine; DNA-PKcs: DNA-PK catalytic subunit; NHEJ: non-homologous end joining; HR, homologous recombination; KEAP1, Kelch-like ECH-associated protein 1; Nrf2, nuclear factor E2-related factor 2; NFE2L2, nuclear factor, erythroid derivative two like 2; bZIP, basic leucine zipper; ARE, antioxidant response; ROS, reactive-oxygen species; Prdx1, peroxiredoxin 1; HO-1, heme oxygenase-1; SOD-1, superoxide dismutase-1; NQO1, NAD(P)H dehydrogenase quinone 1; GCLM, glutamate-cysteine ligase modifier; GSTs, glutathione S-transferases; VEGF, vascular endothelial growth factor; TGF-α, transforming growth factor-alpha; EGFR, epidermal growth factor receptor; ORR, objective response rate; NCCN, National Comprehensive Cancer Network; PFS, progression-free survival; CI, confidence interval; BER, base excision repair; SSBs, single-strand breaks; DSBs, double-strand breaks; PARP, poly (ADP-ribose) polymerase; HRD, homologous recombination deficiency; 5mC, DNA 5-methylcytosine; DNMT, DNA methyltransferases; RET, Ret Proto-Oncogene; RTK, receptor tyrosine kinase; ABL1, ABL proto-oncogene 1, non-receptor tyrosine kinase; SIRT1, silent information regulator T1; AMPK, AMP-activated protein kinase; FDA, Food and Drug Administration; IHC, immunohistochemistry; qPCR, quantitative polymerase chain reaction; 2DG, 2-deoxy-d-glucose

## Glossary

**Table:**

HLRCC	hereditary leiomyomatosis and renal cell carcinoma
RCC	renal cell carcinoma
FH	fumarate hydratase
LRCC	leiomyomatosis and renal cell cancer
WHO	Worldwide health organization
VEGFR	vascular endothelial growth factor receptor
TKIs	tyrosine kinase inhibitors
mTOR	mammalian target of rapamycin
TCA	tricarboxylic acid
HIF	hypoxia-inducible factor
PHD	prolyl hydroxylase
EPO	erythropoietin
EGF	epidermal growth factor
TGF-β3	transforming growth factor beta3
GLUTs	glucose transporters
O2	oxygen
2-OG	2-oxoglutarate
ODD	oxygen-dependent degradation domain
pVHL	Von Hippel-Lindau tumor suppressor protein
Fe2+	Ferrous iron
Fe3+	Ferric form
EMT	Epithelial-mesenchymal transition
TET	Ten-Eleven Translocation
5-hmC	5-hydroxymethylcytosine
DNA-PKcs	DNA-PK catalytic subunit
NHEJ	non-homologous end joining
HR	homologous recombination
KEAP1	Kelch-like ECH-associated protein 1
Nrf2	nuclear factor E2-related factor 2
NFE2L2	nuclear factor, erythroid derivative two like 2
bZIP	basic leucine zipper
ARE	antioxidant response
ROS	reactive-oxygen species
Prdx1	peroxiredoxin 1
HO-1	heme oxygenase-1
SOD-1	superoxide dismutase-1
NQO1	NAD(P)H dehydrogenase quinone 1
GCLM	glutamate-cysteine ligase modifier
GSTs	glutathione S-transferases
VEGF	vascular endothelial growth factor
TGF-α	transforming growth factor-alpha
EGFR	epidermal growth factor receptor
ORR	objective response rate
NCCN	National Comprehensive Cancer Network
PFS	progression-free survival
CI	confidence interval
BER	base excision repair
SSBs	single-strand breaks
DSBs	double-strand breaks
PARP	poly (ADP-ribose) polymerase
HRD	homologous recombination deficiency
5mC	DNA 5-methylcytosine
DNMT	DNA methyltransferases
RET	Ret Proto-Oncogene
RTK	receptor tyrosine kinase
ABL1	ABL proto-oncogene 1, non-receptor tyrosine kinase
SIRT1	silent information regulator T1
AMPK	AMP-activated protein kinase
FDA	Food and Drug Administration
IHC	immunohistochemistry
qPCR	quantitative polymerase chain reaction
2DG	2-deoxy-d-glucose
